# Colonization-Competition Tradeoffs as a Mechanism Driving Successional Dynamics in Ectomycorrhizal Fungal Communities

**DOI:** 10.1371/journal.pone.0025126

**Published:** 2011-09-19

**Authors:** Peter G. Kennedy, Logan M. Higgins, Rachel H. Rogers, Marjorie G. Weber

**Affiliations:** 1 Department of Biology, Lewis & Clark College, Portland, Oregon, United States of America; 2 Department of Ecology and Evolutionary Biology, Cornell University, Ithaca, New York, United States of America; University of Tartu, Estonia

## Abstract

Colonization-competition tradeoffs have been shown to be important determinants of succession in plant and animal communities, but their role in ectomycorrhizal (ECM) fungal communities is not well understood. To experimentally examine whether strong spore-based competitors remain dominant on plant root tips as competition shifts to mycelial-based interactions, we investigated the mycelial competitive interactions among three naturally co-occurring ECM species (*Rhizopogon occidentalis*, *R. salebrosus*, and *Suillus pungens*). Each species was grown alone and in all pair-wise combinations on *P. muricata* seedlings in experimental microcosms and culture assays. Competitive outcomes were assessed from ECM root tip colonization, soil mycelial abundance, and mycelial growth in culture. In the microcosm experiment, we observed a clear competitive hierarchy of *R. salebrosus*>*R. occidentalis*>*S. pungens*. Competitive effects were also apparent in the culture assays, however, no similar hierarchy was present. These results contrast with our previous findings from spore-based competition, suggesting that ECM competitive outcomes can be life-stage dependent. The differing competitive abilities observed here also showed general correspondence with patterns of ECM succession in *Pinus muricata* forests, indicating that competitive interactions may significantly influence temporal patterns of ECM community structure.

## Introduction

One of the longest and most well studied patterns in ecology is the change in community composition through time [1], [2], [3]. In many communities, there is a sequential set of transitions among different species following colonization of a new environment [4]. This ordered change, known as succession, is caused by a variety of processes including tradeoffs in species traits, changing environmental conditions, and interactions among different trophic levels [5], [6]. The majority of the succession literature has focused on plant and animal communities (e.g. [7], [8], [9]), however, advances in molecular identification techniques [10], [11] have resulted in a number of recent investigations of succession in microbial communities ([12] and references therein). The microbial studies show general similarities to those observed for larger organisms, indicating that successional dynamics appear to be a consistent feature across all the major domains of life [13].

Ectomycorrhizal (ECM) fungi represent a dominant group of eukaryotic microorganisms in many forest soils and play an essential role in plant growth, nutrient cycling, and food web dynamics [14], [15]. Like many other ecological guilds, patterns of succession in ECM communities have been documented in multiple study systems, based on both fruit body (i.e. mushroom) surveys [16], [17], [18] and assessment of colonized root tips [19], [20], [21]. Currently, however, the determinants of ECM succession are not well understood. One factor known to be important in plant community succession is a trade-off between colonization and competition ability [8]. For ECM fungi, a colonization-competition tradeoff would involve either allocating resources to vegetative structures such as mycelia, which are essential for colonizing new root tips and competing for soil resources, or to dispersal structures such as fruiting bodies and their associated spores. Peay et al. [22] examined ECM colonization-competition relationships in an experimental system where a major fire created a series of *Pinus muricata* ‘tree islands’ that differed in both size and distance from an unburned forest. They found that species that occurred widely and colonized small islands tended to be those that invested the most in dispersal structures relative to vegetative structures. For example, *Suillus pungens*, which occurred on every island surveyed, was found in 43% of fruit body samples but only 13% of root tip samples, while *Russula amoenolens*, which was found only on the largest islands, was present in only 23% of fruit body samples, but 35% of root tip samples. Although this observational data is suggestive of a colonization-competition tradeoff, additional experimental studies are needed to better link variation in ECM species traits with field distribution patterns.

Over the last twenty years, there has been considerable progress in understanding the ecological importance and mechanisms of ECM competition ([23] and references therein). We have previously shown that “priority effects” (i.e. the preemptive colonization of resources resulting in a competitive advantage) are particularly important among a suite of *Rhizopogon* species, which are co-dispersed by mammal mycophagy and strong competitors in U.S. *Pinus muricata* forests [24], [25]. *R. occidentalis* is typically the competitive superior to *R. salebrosus* on *P. muricata* seedlings when both are inoculated by spore [24], [26], but, in the mature *P. muricata* forests, *R. salebrosus* is the dominant *Rhizopogon* species present and *R. occidentalis* is largely absent ([27],T. Bruns, pers. com.). Given these results, how does the putatively competitive inferior (i.e. *R. salebrosus*) become the more abundant species during ECM succession? Kennedy et al. [24] suggested that one explanation may involve differences in spore- versus mycelial-based competitive abilities. This scenario was based on the fact that *R. salebrosus* can occupy the majority of colonized root tips on seedlings where both *Rhizopogon* species are present [24], [28] despite the observation that *R. salebrosus* is generally excluded from colonizing *P. muricata* seedlings due to slower spore germination relative to *R. occidentalis* [26]. Those results suggested that if priority effects associated with spore-based competition are leveled (i.e. both species colonized the seedling early on), *R. salebrosus* may be a stronger competitor at the mycelial stage. This speculation also matched larger patterns seen in other ECM systems, where early successful species are rapid root tip colonists from spore and later successful species are better colonists from mycelium [16], [29].

To experimentally examine whether differences in mycelial competitive ability may reconcile the differences between observed ECM distribution patterns in *P. muricata* forests and our earlier results on spore-based competitive ability, we investigated the mycelial competitive interactions among *R. occidentalis*, *R. salebrosus*, and *Suillus pungens*. We included *S. pungens* because (i) it is one of the dominant colonizers of young *P. muricata* individuals along with these two *Rhizopogon* species [22] and (ii) it is also present in mature *P. muricata* forests, although not abundant belowground [27]. To assess the mycelial competitive interactions among these species, each was grown alone and in all pair-wise combinations in experimental microcosms on *P. muricata* seedlings and in culture assays. If the competitive interactions observed in this study are distinct from our previous results [24], [26], we can infer fungal life stage (i.e. spore vs. mycelium) may be a critical determinant of competitive success and also play an important role in ECM successional dynamics.

## Methods

### Study system and microcosm experiment

Cones of *P. muricata* were collected from multiple trees at Point Reyes National Seashore in June 2007. Soil was collected in March 2007 from a mixed scrub-grassland site at Point Reyes National Seashore (38°11.807′ N, 122°57.736′ W). The soil is classified as a Kehoe variant coarse sandy loam, which is a deep, well-drained soil derived from quartz-diorite bedrock moraine. This site was selected because the soil is very similar to that present in *P. muricata* forests, but was previously found to have quite low EMF inoculum [30]. To eliminate the remaining inoculum, soil was autoclaved at 121 degrees C for one hour on two consecutive days. Multiple fruit bodies of *R. salebrosus*, *R. occidentalis*, and *S. pungens* were collected from Point Reyes during fall 2007. Spores of *Rhizopogon* species were obtained by macerating fruit bodies in a blender with 500 ml of distilled water for 1 minute and then passing the resulting slurry through cheese cloth to separate fruit body fragments from the spores. *S. pungens* spores were collected by placing caps from multiple fruit bodies on wire mesh suspended over a tin foil wrapped collection container for 48 hours. Spores were removed from the tinfoil, added to distilled water, and quantified using a haemocytometer. Spores of each species were sprayed onto individual aliquots of autoclaved soil for seedling inoculum (see below). The inoculated soils were then mixed 1∶1 (v/v) with coarse sand to reduce compaction and stored at 8 degrees C. All necessary permits were obtained for the described field studies (Pt. Reyes National Seashore permit #: PORE-2002-SCI-0031).

**Figure 1:**
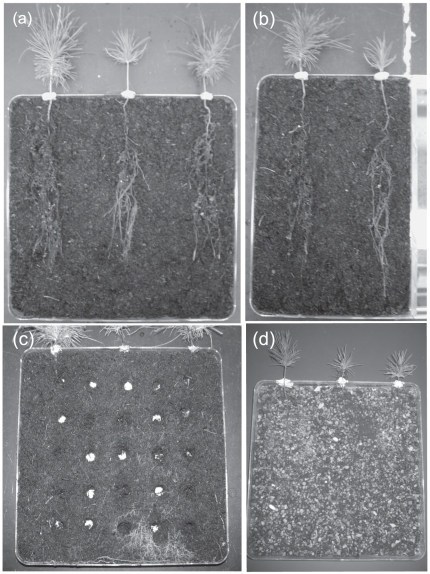
Photographs of the microcosm experiment. (a) In the two-species treatments, the side *Pinus muricata* seedlings were pre-colonized by different ectomycorrhizal (ECM) species and the middle seedling was initially uncolonized by ECM fungi. (b) The single-species treatment was designed identically, but with only one pre-colonized seedling. (c) To assess soil mycelial abundance in the two-species treatments, soil samples were taken from different locations within the microcosm. The microcosms pictured in (a), (b), and (c) came from a pilot experiment in which peat moss was used as the soil substrate. (d) One of the microcosms in the main experiment, which used field soil mixed with coarse sand. Note only nine soil samples were taken in the full experiment, not twenty-five as pictured in the pilot experiment.

In March 2008, three surface-sterilized *P. muricata* seeds were planted into individual 160 ml “ray leach” cone-tainers (Steuwe and Sons, Corvallis, OR, USA) containing 100 ml of inoculated (with one of the three species) or uninoculated soil. Seedlings were grown in a growth chamber under saturated moisture conditions and after one month cone-tainers were weeded to contain a single *P. muricata* individual. The chamber was set at a light intensity of ∼220 µmol m^−2^ s^−1^, a 14 : 10 hour light : dark cycle, with temperatures ranging from 18 – 20 degrees C. In June 2008, seedlings were removed from cone-tainers and their root systems were washed extensively to remove adhering soil. This washing ensured that the competitive interactions described below would be based on mycelial rather than spore-based colonization. Seedlings were then transplanted into flat Petri plate microcosms (243×243×18 mm, Nunc Brand Products, Naperville, IL, USA) containing ∼350 ml of uninoculated soil. In the two-species treatment, three seedlings were planted ∼6 cm equidistant from one another (Figure 1a). The two side seedlings were colonized by ECM species in pair-wise combinations; *R. salebrosus* and *R. occidentalis*, *R. salebrosus* and *S. pungens*, *R. occidentalis* and *S. pungens*. Immediately prior to transplanting, all ECM colonized seedlings were visually scored on a 1–5 scale and assigned to microcosms based on as similar levels of colonization as possible (in nearly all cases the pairs were in the same category, occasionally they differed by one category). The middle seedling in all the two-species treatments was uncolonized by ECM fungi at the time of transplant. Single-species treatments with one colonized and one non-ECM seedling were set up in the same way, however, the overall size of the microcosms was constrained on one side to mimic the area present in the two-species treatments (Figure 1b). The microcosms were maintained under non-sterile conditions at saturated soil moisture levels for five months. Twelve replicates were established for each treatment (N = 72).

In November 2008, the microcosms in which all seedlings survived were harvested to assess ECM colonization (see Table 1 for final N). Each seedling was individually removed from its microcosm and soil was gently rinsed from its root system. Under a 10x dissecting microscope, all live ECM root tips were removed. Bulked ECM root tips from each seedling were flash frozen in liquid nitrogen and lyophilized. The remaining portion of the root system was oven-dried at 60°C for 72 hours. Samples were individually weighed and ECM colonization was calculated as (ECM root biomass/(ECM root biomass+non-ECM root biomass))*100. This metric, % ECM biomass, is not equivalent to other measures of % ECM colonization that involve root tip counting. However, due to high similarities in ECM morphology across these three species, this metric allows for clear comparisons of competitive ability (see [25] for additional discussion of this metric).

**Table 1:** Colonization of the initially non-ectomycorrhizal (ECM) seedlings in the microcosm experiment.

Competition	Species		Proportion
treatment	combination	N	colonized
Single	*R. salebrosus* only	6	0.83
Single	*R. occidentalis* only	8	0.75
Single	*S. pungens* only	6	0.50
Two	*R. salebrosus-R. occidentalis*	6*	1.00
Two	*R. salebrosus-S. pungens*	8	1.00
Two	*R. occidentalis-S. pungens*	8	1.00

*In the two-species *R. salebrosus–R. occidentalis* treatment, six of the initially non-ECM seedlings were colonized, however, due to a processing error during lyophilization resulted in ECM tips from only three of those seedlings being available for molecular identification.

Our primary interest in the two-species treatments was to examine which ECM species colonized the initially non-ECM seedling in the middle of the microcosms. By assessing this colonization, we could determine which ECM species had more effective mycelial spread from already colonized root tips. Since these three species have very similar ECM root tip morphologies, we used molecular identification to determine patterns of colonization on the middle seedlings. Total genomic DNA was extracted from 20 random ECM root tips per seedling using a REDExtract-N-Amp Plant kit (Sigma-Aldrich, St. Louis, MO, USA). This sample size was previously determined to accurately represent competitive outcomes on individual seedlings in this system [25]. DNA from single root tips was extracted and the internal transcribed spacer (ITS) rRNA gene region was PCR amplified using the fungal-specific primer pair ITS1F and ITS4 under conditions previously described [25], [31]. RFLP digests of PCR products were conducted using the restriction enzyme *Cfo*1, which clearly distinguishes the three species (P. Kennedy and A. Lundgren, unpublished data). Digests were visualized on 2%/1% agarose gels and banding patterns of each species were determined by eye. The ratio of ECM root tips belonging to each species was multiplied by the total EMF root biomass of each seedling to calculate respective species' biomasses. Since the focus of this study was on determining the identity of the colonization of only the middle seedlings, no molecular analyses on the side seedlings were conducted.

### Soil mycelial quantification

To analyze the soil mycelial abundance of each species in the two-species microcosms, small soil samples (∼1 g) were taken from nine locations prior to removing the seedlings (Figure 1c). Samples were immediately flash frozen in liquid nitrogen and stored at -20°C. Just before DNA extraction, samples were unfrozen, examined under a 10X dissecting scope, and any root fragments were removed. DNA was extracted using a MoBio UltraClean Soil DNA kit following manufacturer's instructions for maximum DNA yields.

We used SYBR real-time PCR to quantify the amount of mycelium present. Species-specific primers were designed for *R. salebrosus* (forward: 5′-GAC CTA TGT CTT CGT AAC-3′, reverse: 5′-TTA TCT CAA AGA ACC GC-3′), *R. occidentalis* (forward: 5′-GCT TTC TAG TTA AAG TCT AGG-3′, reverse: 5′-GAC TAC TGA TAG TCG TCG-3′), and *S. pungens* (forward: 5′-CGA GGG AAA GGC GGA GAG CTG TAG-3′, reverse: 5′-CCA TAC GGC GAA AAG TCC GGA AGA G-3′) by visually comparing the ITS alignments of these three species (their specificity with respect to other ECM species was not explicitly tested). Primers were tested for *in vivo* specificity among the three test species, primer dimmer formation, and optimized following manufacturer's recommendations [32]. All real-time PCR reactions were run on an iQ5 thermocycler (Bio-Rad, Hercules, CA, USA) with each 15 µL reaction containing 7.5 µl of 2x SYBR IQ Supermix, 0.15 µl of each 50 mM primer, 1 µL of 1∶100 diluted sample DNA, and 6.2 µL of sterile de-ionized water. Thermocycling conditions were 95°C for 3 minutes, followed by 34 cycles at 95 degrees C for 10 seconds and 30 seconds at annealing temperatures of 55, 49, 49 degrees C for *R. salebrosus*, *R. occidentalis*, and *S. pungens*, respectively. All samples were initially run in duplicate per species and any samples with greater that 0.5 difference in C_t_ values (the level at which template fluorescence exceeds background fluorescence) between replicates were re-run. If differences remained above 0.5 in the second run, those samples were excluded from final analyses.

Attempts to determine the exact (rather than relative) amount of mycelium per unit soil (*sensu* [33]) were unsuccessful due to high inter-replicate variability across a range of soil standard quantities. As such, we compared the relative abundance of mycelia from each species using direct comparisons of C_t_ values. Because ITS copy number varies across fungal species [34], the same C_t_ values among different species do not necessarily correspond with identical numbers of ITS copies. To account for this discrepancy, we calculated the individual range of C_t_ values from each species and grouped samples into the following categories: *R. salebrosus* (RS) high = 20.14−24.53, RS medium = 24.54−28.93, RS low = 28.94−33.32, RS none = no C_t_ detected, *R. occidentalis* (RO) high = 26.16−28.22, RO medium = 28.23−30.28, RO low = 30.29−32.34, RO none = no C_t_ detected, *S. pungens* (SP) high = 22.81−25.68, SP medium = 25.69−28.54, SP low = 28.55−31.41, SP none = no C_t_ detected. Based on their C_t_ value, samples were then assigned one of the following abundance categories: 3 = high, 2 = medium, 1 = low, 0 = none.

### Culture experiment

To further examine mycelial competitive interactions among *R. salebrosus*, *R. occidentalis*, and *S. pungens*, each species was also grown in single- and two-species pure culture assays. Isolates of each species were obtained from individual fruit bodies collected from Point Reyes and cultured on modified Melin Norkrans (MMN) media (glucose 5.0 g,, malt extract 2.0 g, potassium phosphate monobasic [KH_2_PO_4_] 0.5 g, ammonium phosphate dibasic [(NH_4_)2HPO_4_] (10% solution), 2.5 ml magnesium sulphate [MgSO_4_] 0.15 g, calcium chloride [CaCl_2_] (1% solution) 5 ml, sodium chloride [NaCl] (1% solution) 2.5 ml, ferric chloride [FeCl_3_] (1% solution) 1.2 ml, agar 15 g, water 1 L). For the competition assays, two isolates of each species were selected and grown in duplicate in all single and pair-wise isolate combinations. Plugs from four-week old cultures served as initial inoculum and were placed at the center (single-species treatment) or equidistant locations (two-species treatment) on 9 cm diameter Petri dishes. Cultures were maintained in the dark at room temperature (∼24°C). Growth was recorded every seven days and stopped once cultures reach the edge of the dish or there was no further growth the following week. The area of the plate occupied by mycelium of each species (minus the original plug) was calculated using the software program ImageJ. Since growth rates varied across isolates and species, final area was divided by total number of weeks so that mycelial growth was expressed as cm^2^/week.

### Defining competition and statistical analyses

We define competitive effects as a decrease in the growth of one EMF species in the presence of another EMF species. While this definition has inherent limitations (see [23] for a thorough discussion of this topic), it emphasizes comparisons between the single- and two-species treatments common to both the microcosm and culture experiments. We also differentiate between two main types of competition; interference competition, which involves direct competitive interactions (e.g. takeover of occupied root tips or soil/agar), and exploitation competition, which involves indirect interactions through the depletion of a common resource [4]. In this case, we see space as the primary resource for which the ECM fungi are competing, either on seedling roots and occupation of soil/agar.

One-way fixed-factor ANOVAs were used to assess competitive outcome in the microcosm and culture experiments. For each species, % EMF biomass and mycelial growth rate were compared among the single- and two-species treatments. Prior to each ANOVA, variances were assessed and data transformations (arcsine or log) were conducted to reduce variance heterogeneity where necessary. Post-hoc Tukey HSD tests were performed to determine significant differences among treatment means. To examine mycelial abundance patterns in the microcosm experiment, the mean abundance score of each soil sample location was calculated. For the culture experiment, a growth ratio value was calculated for each replicate by dividing the mean growth rate during the second half of the experiment by the mean growth rate in the first half of the experiment. Means and standard deviations of those growth ratios were then calculated for each treatment. All analyses were conducted in JMP 5 (SAS Inc., Cary, NC, USA) and considered significant at the 0.05 alpha level.

## Results

**Figure 2:**
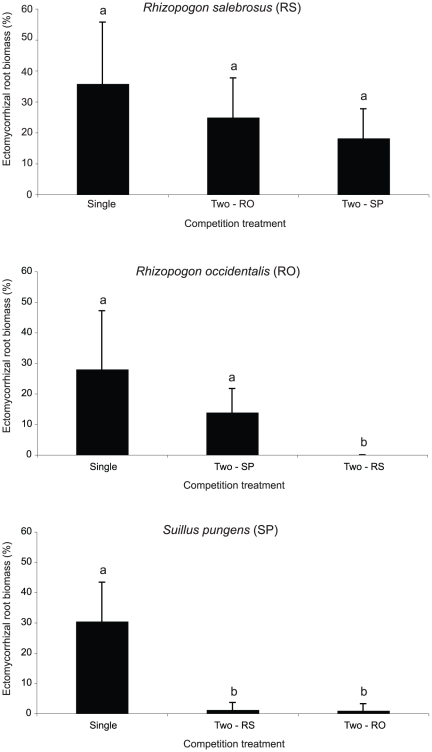
Outcome of ectomycorrhizal (ECM) competition. Bars represent the ECM biomass (% of total root mass) of *Rhizopogon salebrosus*, *R. occidentalis*, and *Suillus pungens* on initially uncolonized *P. muricata* seedlings in the microcosm experiment (mean±one standard deviation). Different letters indicate significant differences among competition treatment means as determined by post-hoc Tukey tests. The results indicate a clear competitive hierarchy of *R. salebrosus*>*R. occidentalis*>*S. pungens*.

In the microcosm experiment, all of the initially non-ECM seedlings (i.e. the middle seedlings) were colonized in the two-species treatments, but colonization patterns for the same seedlings in the single-species treatments were more variable (Table 1). *R. salebrosus* and *R. occidentalis* both colonized the majority of non-ECM seedlings in their single-species treatments, but only half of the non-EMF seedlings were colonized by *S. pungens*. ECM composition of the initially non-EMF seedlings in the two-species treatments varied by species pairing. In both the *R. salebrosus* – *R. occidentalis* and *R. salebrosus*–*S. pungens* pairings, *R. salebrosus* was the dominant colonizer. The % ECM biomass of *R. salebrosus* on the middle seedlings was not significantly different from that on the initially non-ECM seedlings in its single-species treatment (*R. salebrosus* (RS) ANOVA: F_2,15_ = 2.10, P = 0.168, both Tukey tests P>0.05, Figure 2). In contrast, the % ECM biomass of *R. occidentalis* and *S. pungens* on the middle seedlings in the *R. salebrosus* pairings was significantly lower than on the initially non-ECM seedlings in their respective single-species treatments (Tukey tests: RO vs. RS–P<0.05, SP vs. RS–P<0.05, Figure 2b,c). In the *R. occidentalis*–*S. pungens* pairing, *R. occidentalis* was the dominant colonizer of the middle seedlings. The % ECM biomass of *R. occidentalis* on the middle seedlings in the *R. occidentalis*–*S. pungens* pairing was not significantly different from that in its single-species treatment (*R. occidentalis* (RO) ANOVA: F_2,15_ = 5.49, P = 0.016, Tukey test: RO vs. SP–P>0.05), whereas the % ECM biomass of *S. pungens* was significantly lower in the two- than single-species treatment (*S. pungens* (SP) ANOVA: F_2,16_ = 41.51, P<0.001), Tukey test: SP vs. RO–P<0.05).

**Figure 3:**
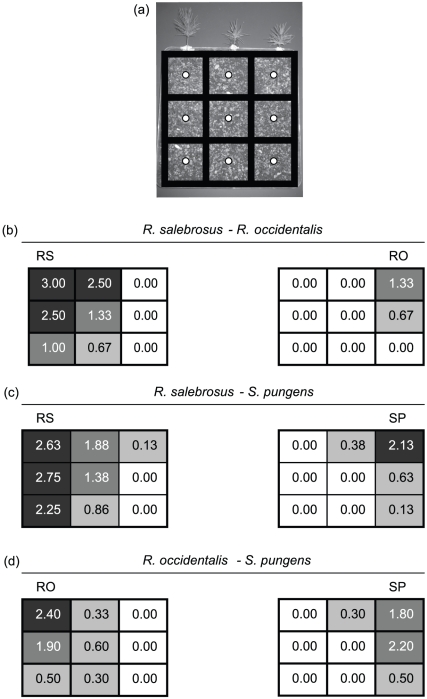
Soil mycelial abundances in the two-species treatments. (a) One of the experimental microcosms with the location of the nine soil samples represented by white circles. The black grid over the microcosm matches the gridded abundance values presented in b-d. (b-d) Soil mycelial abundances of each species in the two-species treatments based on SYBR real-time PCR. Values represent mean category scores (see methods for details); categories were 3 = high, 2 = medium, 1 = low, 0 = no mycelium detected. To better illustrate abundance patterns, locations are color-coded as follows: 3-2.01 = dark grey, 2−1.01 = medium gray, 1−0.01 = light gray, 0 = white. The locations of the pre-colonized seedlings are indicated by the species initials over one side of the microcosm. Soil mycelial abundances reflect the same competitive outcomes as observed in Figure 2.

The patterns in soil mycelial abundance in the two-species treatments followed very similar trends to those seen with ECM colonization of the middle seedling (Figure 3). In the *R. salebrosus–R. occidentalis* and *R. salebrosus–S. pungens* pairings, the soil mycelium in the three vertical middle samples (i.e. those under the middle initially non-ECM seedlings) was dominated by *R. salebrosus*. Soil mycelial quantities of *R. salebrosus* in those samples were somewhat lower than quantities present in the three samples under the initially *R. salebrosus*-colonized seedlings. Soil in the three vertical middle samples in the *R. occidentalis*–*S. pungens* pairing was primarily colonized by *R. occidentalis*. The overall mycelial quantities in the *R. occidentalis*–*S. pungens* middle samples were lower than those present in either of the *R. salebrosus* pairings. In all cases, the side on which the ECM-colonized seedlings were transplanted was dominated by mycelium of that species. In only one pairing, *R. salebrosus*–*S. pungens*, was the mycelium of a species ever detected on the opposite side from which it was planted. In that case, which occurred in a single microcosm, *R. salebrosus* co-occurred with *S. pungens* in one sample at very low abundance (Figure 3b).

**Figure 4:**
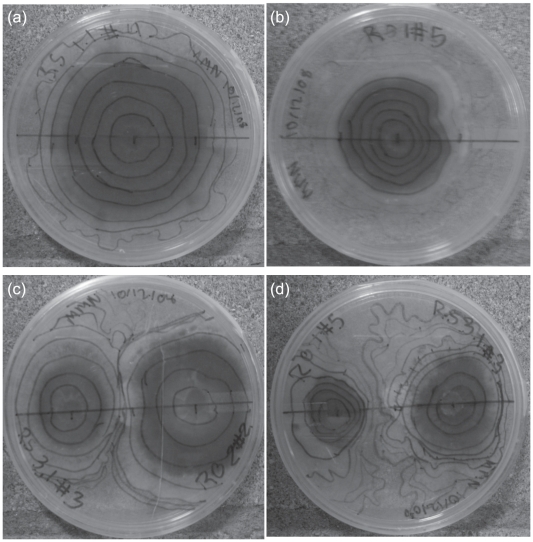
Photographs of the pure culture experiment. (a) A replicate of the *R. salebrosus* single-species treatment. (b) A replicate of the *R. occidentalis* single-species treatment. (c-d) Two replicates of the *R. salebrosus*–*R. occidentalis* two-species treatment, with different *R. occidentalis* isolates. The lines within each dish represent weekly growth traces.

Mycelial growth rates of *R. salebrosus*, *R. occidentalis*, and *S. pungens* in the culture experiment showed greater variation by competition treatment than species identity (Table 2). For all three species, the growth rates were higher in the single- than two- species treatments. That difference was significant for *R. salebrosus* and *S. pungens* (RS ANOVA: F_2,17_ = 21.77, P<0.001; SP ANOVA: F_2,17_ = 20.33, P<0.001), but not for *R. occidentalis* (ANOVA: F_2,17_ = 2.48, P = 0.114), which had higher intra-isolate variation. The lower growth rates in the two-species treatment were putatively attributable to a slowing in growth when the mycelia of the two species sensed one another. This is evidenced by comparing mycelial growth rate ratios for the second and first half of the experiment in the single- and two-species treatments, respectively (Table 2). In the single-species treatments, all three species have mean ratios well above one, indicating that mean growth rates in the second half of the experiment were greater than in the first. In contrast, in the two-species treatments, the mean ratios are all relatively close to one, indicating mean growth rates were approximately the same throughout the experiment. Furthermore, all of the standard deviations in the two-species treatments overlap one and many of the growth rate ratios were actually less than one (i.e. mean growth rates were slower in the second half of the experiment than in the first half). Accompanying this slowing was a complete cessation of growth when the mycelia of competitor species actually met; no growth of one species over the top of another was ever observed before the end of the experiment (Figure 4).

**Table 2:** Mycelial growth rates in the pure culture experiment.

Species	Treatment	Growth (cm^2^/wk)	Growth Ratio
***R. salebrosus*** ** (RS)**	**Single**	**5.13 (1.54) a**	**1.64 (0.20)**
*R. salebrosus*	Two-RO	2.45 (0.36) b	1.18 (0.32)
*R. salebrosus*	Two-SP	2.15 (0.56) b	1.15 (0.31)
***R. occidentalis*** ** (RO)**	**Single**	**4.73 (3.84) b**	**2.03 (0.28)**
*R. occidentalis*	Two-SP	2.17 (0.74) b	1.20 (0.31)
*R. occidentalis*	Two-RS	2.42 (1.42) b	1.24 (0.25)
***S. pungens*** ** (SP)**	**Single**	**4.02 (0.34) a**	**1.55 (0.24)**
*S. pungens*	Two-RS	2.36 (0.65) b	1.16 (0.23)
*S. pungens*	Two-RO	2.23 (0.34) b	1.04 (0.24)

Growth rates are grouped by species and competition treatment, with the single-species treatment in bold. Values are means +/- 1 standard deviation in parentheses. Different letters indicate significant differences in growth rates as determined by post-hoc Tukey HSD tests. Growth ratios were calculated by dividing the mean growth rate in the second half of the experiment by the mean growth rate in the first half of the experiment.

## Discussion

The outcomes of the microcosm and culture experiments indicated that mycelial interactions among *R. salebrosus*, *R. occidentalis*, and *S. pungens* can be strongly competitive, and the dominance of *R. salebrosus* in the microcosm experiment suggest that the initial competitive advantage that *R. occidentalis* gained from having faster germinating spores than *R. salebrosus* [24], [26] can be overcome once the competitive interactions among these two species are based on mycelium. These new mycelium-based results also help explain the initially spore-based competitive reversal we had observed between *R. salebrosus* and *R. occidentalis* in a previous experiment [28]. There we noted that high root densities likely resulted in greater spore-based colonization by *R. salebrosus*, which combined with better mycelial competitive abilities observed here, would lead *R. salebrosus* to be the competitive superior. More broadly, the data in this study, taken together with our previous work [24], [26], provides the first experimental evidence that changes in abundance of two naturally occurring ECM species over successional time scales may be the result of tradeoffs between colonization and competitive ability.

The poor performance of *S. pungens* as a mycelial competitor corresponds well with the observation that it is infrequently found on ECM root tips in the field, even directly below its own fruit bodies [22], [27]. Our *S. pungens* data is also similar to that of *S. luteus* in the study of Wu et al. [35], who found that species to be a relatively weak mycelial competitor against *Pisolithus tinctorius* and an unidentified ECM species, ‘Tanashi 01’ (now known by sequence to belong to the family Atheliaceae, K. Nara, per. com). Based on the root tip abundance of *S. pungens* in mature *P. muricata* forests [27], we expected that it would be a mycelial competitive inferior to *R. salebrosus*, which we did observe, but a competitive superior to *R. occidentalis*, which we did not. Although these results do not directly match with the field distributions of *R. occidentalis* and *S. pungens*, the middle seedling in one of the *R. occidentalis* – *S. pungens* microcosms was completely colonized by *S. pungens*. This suggests that *S. pungens* is not always a competitive mycelial inferior and that some local stochasticity in competitive outcomes may be important in allowing weaker mycelial competitors to persist in mature *P. muricata* forests.

The type of competition occurring among these ECM species was similar in the microcosm and culture experiments. In both settings, there was basically no encroachment into areas occupied by a competitor (e.g. mycelial deadlocks in the pure culture experiment, the general lack of co-colonization of middle seedlings, no co-colonization of individual ECM root tips, and the largely non-overlapping mycelial distributions in the microcosm experiment). This indicates that exploitation rather interference competition was the primary way competitive outcomes were determined among these species. This type of competition places a premium on rapid rates of mycelial spread (to preemptively capture as much resource as possible), which may be particularly well suited for the ‘long-distance’ mycelial exploration types of *Rhizopogon* and *Suillus* species [36], [37]. However, Wu et al. [35] found that mycelial competitive outcomes were strongly more influenced by mycelial architecture than rates of spread. In that study, *P. tinctorius* and ‘Tanashi 01’ had similar rates of mycelial spread, but the spread of the former was relatively patchy, while that of the latter was quite homogenous. They found that openings left in the area initially occupied by *P. tinctorius* were key in allowing ‘Tanashi 01’ to subsequently invade *P. tinctorius* territory. Based on both the form of culture growth (Figure 4) and the soil mycelial abundances (Figure 3), the architectures of mycelial spread of *R. salebrosus*, *R. occidentalis*, and *S. pungens* all appeared to be homogenous, which may explain why so little competitive encroachment was observed in this study. Given these differing results, future studies examining ECM species with a greater range of mycelial spread architectures will be particularly helpful in better understanding the relative importance of exploitation versus interference competitive strategies.

While patterns of ECM succession have been well documented both above- and belowground [16], [17], [18], [20], [21] the mechanisms responsible for those patterns are less well understood. Connell and Slayter [7] outlined three general mechanisms by which communities change over time; (1) facilitation, where the presence of early colonists hastens the colonization of later ones, (2) inhibition, where early colonists retard the colonization of later ones, and (3) tolerance, where the presence of early colonists have no significant effect on later ones. Although the data to discriminate among these mechanisms for ECM fungi is limited, in a previous split-root experiment [25], we observed neither an increase (which would support facilitation) nor a decrease (which would support inhibition) of colonization by *R. salebrosus* (the later colonizing species) when a different part of the same seedling root system was colonized by *R. occidentalis* (the early colonizing species). That result suggests that colonization of available non-ECM roots by later colonizers is not strongly influenced by the species already present. Because new roots are constantly introduced as plants grow and their production seems largely independent of prior ECM colonization, this provides key opportunities for later colonizing species to colonize along side early colonizing species (a situation not possible in Connell and Slatyer's inhibition model). Once both types of colonizers are established on the same root system, data from this study indicate that stronger mycelial competitors become dominant over time. Clearly mechanism-related data from additional systems is needed, but in *P. muricata* forests, the tolerance model appears to be the primary mechanism by which ECM succession proceeds.

Our results have important implications regarding ECM competition and succession, but the experimental approaches used also have clear limitations. The most significant limitation was the testing of competitive outcomes for different fungal life stages in different experiments. While we strongly believe that results based on spore competition in these microcosms would have been similar to previous experiments with comparable root densities [24], [26], our conclusion that fungal life stage strongly affects competitive outcomes is based on that assumption. Another factor is that by spacing the pre-colonized ECM seedlings at considerable distances from the initially non-ECM middle seedlings, we may have overemphasized the importance of rates of mycelial spread in determining competitive success. It is likely that similar distances between colonized and uncolonized host roots exist in natural settings, but in mature *P. muricata* forests, there may be additional uncolonized roots at closer proximities as well (P. Kennedy, pers. obs.). The distance between roots may be particularly important for ECM species with ‘contact’ or ‘short-distance’ mycelial exploration types [36], which are likely strong competitors when root density is high (i.e. inter-root distance is low), but poor when it is low [38]. A third factor was that the microcosm experiment was conducted on *P. muricata* seedlings in a highly simplified system, which obviously did not capture all of the biotic and abiotic changes occurring in natural forests over successional time scales. This difference may help explain the mismatch between our experimental results with *R. occidentalis* and *S. pungens* and their abundances in mature *P. muricata* forests. Finally, the microcosm experiment was done in a growth chamber to avoid potential colonization from other ECM species (a common phenomenon in greenhouse and field studies). While the growth chamber does not capture the biotic and abiotic variation present in a field-based experiment, we have previously found that the outcome of similarly designed ECM competition experiments is analogous under field and growth chamber conditions [24], [26].

In summary, these results are the first to experimentally demonstrate that ECM competitive abilities can be life-stage dependent. Along with our previous spore-based competitive studies, the current mycelial-based data adds further support to the assertion that interspecific competition plays a significant role in ECM interactions [23]. The differing competitive abilities observed here also corresponded with some of the natural changes in species composition associated with ECM succession, indicating that competitive interactions may significantly influence temporal patterns of ECM community structure. Future studies examining colonization-competition tradeoffs in other ECM study systems are clearly needed and will greatly assist in further understanding the long-term successional dynamics of ECM communities.
